# Effects of the Level of Interactivity of a Social Robot and the Response of the Augmented Reality Display in Contextual Interactions of People with Dementia

**DOI:** 10.3390/s20133771

**Published:** 2020-07-05

**Authors:** Yuan Feng, Emilia I. Barakova, Suihuai Yu, Jun Hu, G. W. Matthias Rauterberg

**Affiliations:** 1 Department of Industrial Design, Eindhoven University of Technology, 5600 MB Eindhoven, The Netherlands; J.Hu@tue.nl (J.H.); G.W.M.Rauterberg@tue.nl (G.W.M.R.); 2 Department of Industrial Design, Northwestern Polytechnical University, Xi’an 710072, China; caid@nwpu.edu.cn

**Keywords:** dementia, social robot, human–robot interaction, interactivity, context, engagement, apathy

## Abstract

The well-being of people with dementia (PWD) living in long-term care facilities is hindered due to disengagement and social isolation. Animal-like social robots are increasingly used in dementia care as they can provide companionship and engage PWD in meaningful activities. While most previous human–robot interaction (HRI) research studied engagement independent from the context, recent findings indicate that the context of HRI sessions has an impact on user engagement. This study aims to explore the effects of contextual interactions between PWD and a social robot embedded in the augmented responsive environment. Three experimental conditions were compared: reactive context-enhanced robot interaction; dynamic context-enhanced interaction with a static robot; a control condition with only the dynamic context presented. Effectiveness evaluations were performed with 16 participants using four observational rating scales on observed engagement, affective states, and apathy related behaviors. Findings suggested that the higher level of interactivity of a social robot and the interactive contextualized feedback helped capture and maintain users’ attention during engagement; however, it did not significantly improve their positive affective states. Additionally, the presence of either a static or a proactive robot reduced apathy-related behaviors by facilitating purposeful activities, thus, motivating behavioral engagement.

## 1. Introduction

People with dementia (PWD) in long-term care (LTC) facilities may benefit from appropriate technological solutions that target the improvement of current disengaged lifestyles [1], by motivating intrinsic interests and engaging in meaningful activities [2,3]. Animal-like social robots are one major area that is gradually gaining attention in dementia care. Such robotic pets (such as PARO [4,5,6], a robotic baby seal, AIBO [7], a robotic dog, NeCoRo [8], a robotic cat, Huggable [9] and CuDDler [10], robotic teddy bears, and Pleo [11,12,13], a robotic dinosaur) are designed to provide companionship and social support, motivate communications, help regulate anxiety, depression, and agitation, and they also demonstrate similar positive effects as animal-assisted therapy for improving quality of life [14,15]. These uniquely designed robots are equipped with multiple sensors, designed with cute and inviting appearances, and behave to evoke positive human emotions [16].

Animal-like social robots have been introduced to PWD as reasonable substitutes for real animals to avoid potential safety hazards (e.g., allergies, infection, or injury) and extra workload on caregivers (e.g., cleaning and taking care of the animals) caused by real animals [17,18,19]. Related research looked into comparison effects between robotic pets and other animal-related stimuli (such as plush toys, real animals, and animal videos) to investigate the effectiveness among different stimuli. Tamura et al. [20] evaluated the therapeutic effects of interaction with a robotic dog AIBO to a toy dog on severe dementia users. The findings suggested that the interaction with both an AIBO and the toy dog led to improvements in responsive behaviors, such as looking at, communicating with, and caring for the stimuli. Libin et al. [8] investigated the difference between a robotic cat NeCoRo and a plush cat on PWD’s agitation, affect, and engagement. Results indicated both types of cats held promise for decreasing agitated behaviors and had positive influences on affect and engagement. The findings of the above two studies suggested, although not identically, similar positive effects (e.g., decreased agitated behaviors and improved affect and engagement) were seen with both kinds of stimuli. In a different study [4], Takayanagi et al. compared the interaction of a PARO with a stuffed lion toy with PWD. The results showed significantly more positive changes in emotional expression, self-initiated talk, and active interaction with the PARO than with the lion toy. More recently, Moyle et al. [21] undertook a 10-week cluster-randomized controlled trial to explore the effects between a PARO and a look-alike plush toy. The findings purposed that the same participants had varied positive and negative responses toward the PARO during a long-term evaluation, depending on the facilitation and personal statues. In addition, Marx et al. [22] examined the effects of five different kinds of dog-related stimuli on engagement, including a puppy video, a real dog, a plush dog, a robotic dog, and a dog-coloring activity. The results showed no statistical differences in engagement duration and positive attitudes among all stimuli, except the coloring activity.

The aforementioned literature, although demonstrating promising evidence on animal-like social robots’ roles in enhancing the engagement of PWD, the nature and extent of the evidence supporting the use of social robots are unclear, and there is no consensus in the documented results that confirms that a robot with relatively higher interactivity performs significantly better than a plush toy on user engagement or the affective states of PWD. Therefore, more studies are needed to further explore the effects of animal-like social robots on the well-being of PWD, as well as potential influential factors that could contribute to enhanced engagement.

The rich interaction of designed agents (e.g., social robots), suggested by Frens et al. [23] and Wada et al. [24], is one key factor for motivating the engagement of users. What is more, as most previous human–robot interaction (HRI) research studies engaged independently from the context, recent studies indicate that the context of a HRI session has the potential for influencing user engagement as well [5,25]. Hoffman et al. [26] conducted two separate studies to explore HRI sessions in a shared music-listening and a video-watching experience. The findings showed differences in how users perceived the presented stimuli and their attitudes toward the companion robot when adding a shared context. Participants enjoyed the music more due to the presence of the responsive behaviors of the robot. Moreover, Hendriks et al. [27] performed a study with PWD to investigate whether adding audio–visual contextual cues would contribute to a more engaging play experience with a robotic dinosaur—Pleo. The results indicated an increase in gaze behavior when the context was added. To our best knowledge, none of the existing research has undertaken a comparison study between robot interaction and static robot/toy play within a shared context with PWD. Therefore, in this study, we took the exploration of HRI with the elderly with dementia to a new level—an animal-like social robot was embedded in an augmented responsive environment. Two factors—the level of interactivity of a social robot and context of HRI—that could potentially contribute to the enhanced engagement of PWD were combined.

**Figure 1:**
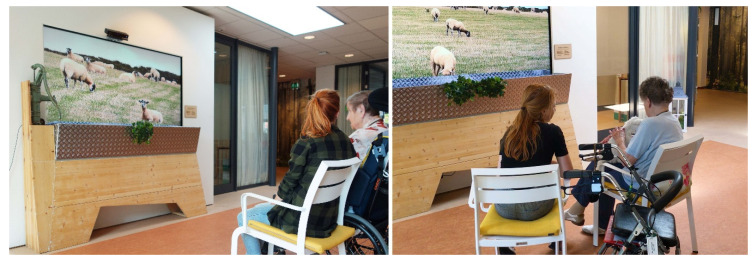
Design LiveNature implemented in Vitalis, including a soft-fur covered robotic sheep and an augmented reality display. The picture on the left shows an example of the interaction session with the control condition, and the picture on the right demonstrated a scenario of a participant interacting with a robotic sheep with response from the augmented reality display.

This study was built on our previous work—an interactive installation design called LiveNature [28]. LiveNature engages PWD in relaxing nature experiences through the combined use of a robotic sheep and an augmented reality display mounted on the wall, shown in Figure 1. The dynamic context was responsive, as the HRI not only triggers the motion and sound feedback of the robot but also the visual–audio responses from the display. The 87-inch ultra-high definition screen display shows the dynamic video content of a grass field with a heard of sheep to simulate a window outlook experience of typical Dutch farm scenery. The virtual content on the screen was augmented using a physical interactive interface (an old-fashioned water pump and an animal feeding trough) [29] and a physical robotic sheep, to reinforce the tactile interaction for the completeness of the multi-sensory engagement. The soft-fur covered robotic sheep is a prototype built through reprogramming a Pleo robot and is weighted similar to a real baby lamb. Since this work was undertaken in collaboration with a Dutch residential home, we addressed several aspects of the design that are familiar to a generation of elderly Dutch people to trigger reminiscence, evoke memories, and emotional responses. LiveNature combined a robotic sheep with an augmented responsive environment to: (1) provide a content-related context for improving acceptability at start of the HRI session; (2) help sustaining attention and interests during engagement by providing multiple feedback from both the robot and the screen-display; (3) create a vivid multi-sensory environment for a calm and relaxing rich interactive experience.

Three experimental conditions were adopted in this study with different levels of interactivity of a social robot and contextual influences from the augmented reality display: Condition 1—reactive context-enhanced interaction with a proactive robot (C1); Condition 2—dynamic context-enhanced interaction with a static robot (C2); a control condition with only dynamic context presented (CC). The robotic sheep that invites interactions and provokes contextualized feedback from the environment was used for C1, and it was turned off as the static robot. Effectiveness evaluations were performed using four observational rating scales on observed engagement, affect, and apathy-related behaviors.

The objectives of this study are:

- To explore the effects of contextual interactions between PWD and an animal-like social robot embedded in the augmented responsive environment in an LTC facility;
- To investigate which experimental condition is more effective in enhancing engagement in PWD, provoking positive affective responses and reducing apathy related behaviors.

Since most existing work regards social robot use focused on the feasibility, acceptability, and effectiveness of the robotic companions on dementia users [30,31], this study fills in the gap of dementia-related research by exploring the impact of the context of interaction on user engagement, and further investigated the factors that could potentially benefit PWD more during HRI sessions.

## 2. Materials and Methods

### 2.1. Participants

**Figure 2:**
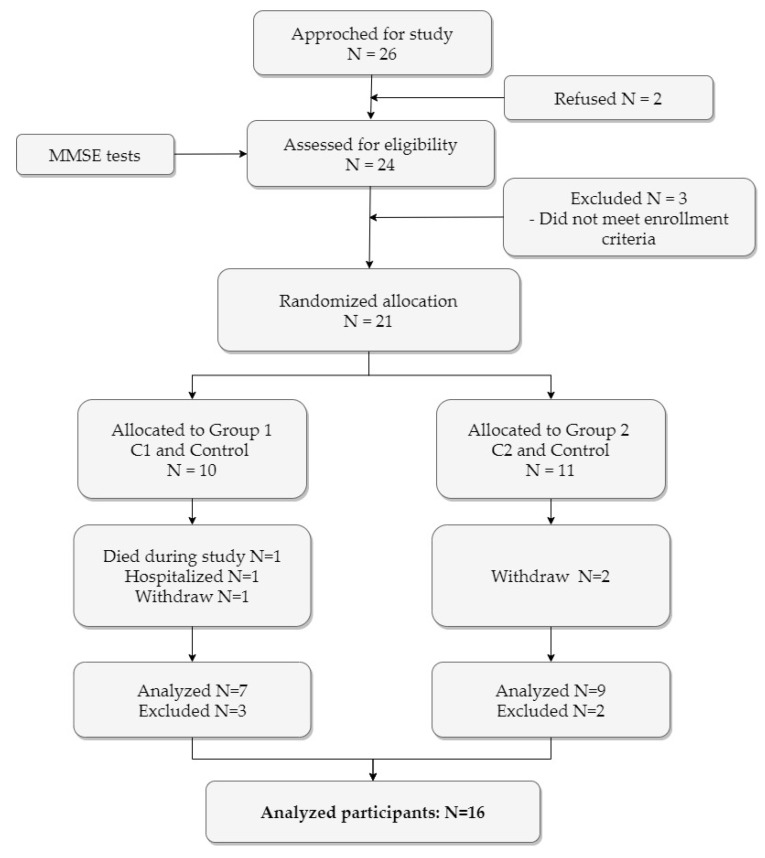
Flow diagram of recruitment, enrollment, allocation, and the number of participants.

Twenty-six residents from a Vitalis WoonZorg Groep LTC center (Vitalis for short), Eindhoven, the Netherlands, where inhabitants with formal diagnoses of dementia live in an enclosed environment, were approached for the study. Informed consent was obtained for twenty-four residents (*N* = 24) and they went through the screening procedures for eligibility. The inclusion criteria were: (1) a documented formal diagnosis of dementia; (2) an age of 65 and above; (3) a Mini-Mental State Examination (MMSE) score lower than 24; (4) a physical ability to sit, hold, and interact with the robotic sheep. The participants with acute visual or auditory impairment reported by staff were excluded from the experiment. The principal investigator contacted the care facility to hold a family meeting pre-experiment with legally authorized representatives of residents for presenting all relevant information regarding the experiment, signing informed consent, and residents’ rights to refuse to participate during any time. The participant recruitment flow is shown in Figure 2.

### 2.2. Study Design

To test the effects of the experimental conditions, 21 eligible participants were allocated to two parallel groups (G1: *n* = 10 and G2: *n* = 11), randomized by living units and stratified by dementia severity. Within each group, participants experienced one experimental condition (G1 participated in C1; G2 participated in C2) and one control condition—CC. Descriptive details of experiment conditions are shown in Table 1. Two sessions of each interaction took place. The interaction sessions were provided once a week and lasted for four weeks in total. Within each group, half of the participants started with the experiment condition and the others with the control condition for eliminating possible confounding factors. In the end, 16 participants’ data were used in the analysis.

All experimental sessions were performed in a real-life setting of the Vitalis living facility. The installation was situated in the public hallway, where two seats were positioned in front of the installation to create a comfortable sitting environment. Experimental sessions took place during non-planned activity time between 10:00 to 12:30 a.m. and 2:00 to 5:00 p.m. with up to 10 sessions planned each day and each session lasting up to 20 minutes. A trained facilitator invited participants one at a time to spend some time to join the session. She was instructed to be inconspicuous while interacting, but would help encourage engagement when needed, and ended the sessions when participants started to lose interest and focus. The facilitator was trained extensively by employing pre-experiment presentations, written guidelines, and was blinded to the objectives of the study. Video and audio materials of all experimental sessions were recorded.

### 2.3. Measures

The MMSE test was administered for eligibility examination before the experiment sessions by the facilitator. The evaluation was carried out using the following validated observational rating scales by blinded raters:

- Observational Measurement of Engagement (OME) [32] was adopted as it is the most widely used scale for assessing the engagement of PWD. It measures engagement through the duration of the time that resident is involved with the stimulus, and level of attention and attitude towards the stimulus on two 7-point Likert scales separately;
- The Engagement of a Person with Dementia Scale (EPWDS) [33] using a 5-point Likert scale was also adopted for evaluating user engagement, as it compensates OME by providing the verbal and social aspects of engagement. EPWDS emphasizes both the social interaction and activity participation (engagement with the stimulus) of PWD across LTC setting. This 10-item scale measures five dimensions of engagement: affective, visual, verbal, behavioral, and social engagement. Each dimension was assessed separately using a positive and a negative subscale, then interpreted collectively to provide an overall impression of engagement. Each item indicates the extent to which the rater agrees or disagrees with the statement (“strongly disagree” = 1, “strongly agree” = 5);
- Observed Emotional Rating Scale (OERS) [34], a 5-point Likert scale for evaluating five affective states: pleasure; anger; anxiety/fear; sadness; general alertness. Items were scored according to the intensity presented during experiment sessions;
- People Environment Apathy Rating Scales–Apathy subscale (PEAR–Apathy subscale) [35], a 4-point Likert scale for assessing apathy related behaviors. It evaluates symptoms of apathy in cognitive, behavioral, and affective domains through six ratings: facial expressions; eye contact; physical engagement; purposeful activity; verbal tone; verbal expression.

The rating scales of OME and OERS were rated on-site through direct observation by an observer after each experiment session. The PEAR–Apathy subscale and EPWDS were rated by a trained research assistant based on the video recordings of the experiment after data collection was completed. Although the rating scales were initially developed for field tests via direct observation, previous research studies also tested the validity and reliability of these tools using videos for indirect observation based ratings [35,36] with positive outcomes. A higher rating score of OME, EPWDS, OERS, or PEAR–Apathy subscale indicates a greater display of a particular effect.

### 2.4. Ethical Considerations

This study was approved by the Board of Vitalis WoonZorg Groep care center, sent on 14 May 2018. All participants (or legal guardians as representatives, when participants were no longer capable of giving consent) gave their informed consent for inclusion before they participated in the study. The research was permitted and conducted in accordance with the requirements of the Eindhoven University of Technology.

## 3. Data Analysis

For the inter–rater reliability (IRR) validity check, a second rater rated and coded partially on the video recordings (13 out of 52 sessions), randomly selected. The IRR between the two coders was calculated using Cohen’s kappa statistic, shown in Table A1, Appendix A. According to Fleiss [37,38], a Kappa value between 0.40 and 0.60 was considered a fair agreement, between 0.60 and 0.75 a good agreement, and above 0.75 an excellent agreement. The data rated by the raters (onsite observer and offsite research assistant) were used for further analysis. Data entry and analysis of all observational rating scales were completed using IBM SPSS Statistics, Version 24. We examined data from OERS and found a very low occurrence of items “Anger”, “Anxiety/Fear”, and “Sadness” and, therefore, they were merged as a single item “Negative Affect” [39]. Differences among the control and experiment conditions were compared using non-parametric statistical tests (Kruskal–Wallis H with pairwise comparisons) for categorical ordinal variables. The analysis of variance (ANOVA) with post hoc tests for continuous variables (item Duration in OME, and all items in EPWDS) were used. Significant alpha was set at *p* < 0.05.

## 4. Results

### 4.1. Participant Demographics

Demographic information was collected from documented medical records of each participant. The 16 analyzed participants were within the age range of 78–92 (*M* = 85.2, *SD* = 4.8) and were at various stages of dementia, according to staff reports. Sample characteristics were summarized as the means and standard deviations (SD) of the continuous variables and as frequencies and percentages of the categorical ones. The demographic of the participating residents, according to group assignment, is described in Table 2. The *t*-test showed no significant difference between the characteristics of G1 and G2.

### 4.2. Results of the Observational Rating Scales Analysis

Results of all rated items from each observational rating scale were summarized using the means and SD, shown in Table A2, Appendix B.

According to the results of OME, a significant difference was found on item “Attitude—Most of the time” with a Kruskal–Wallis test among three conditions (Chi-square = 6.41, *p* = 0.041, *df* = 2). Furthermore, pairwise comparisons of experiment conditions showed “Attitude—Most of the time” was significantly higher when interacting with C1 than C2 (*p* = 0.049), indicating that the participants demonstrated more positive attitude with a higher level of interactivity with the social robot, accompanied by the response from the augmented reality display. No significance was found with the rest of the rating items. The average mean of “Duration” and “Attitude” (both “Most of the time” and “Highest level”) are the lowest during the interaction with C2, meaning the participants were the least engaged when the robot was presented but turned off, compared to the other two conditions.

In terms of EPWDS, statistical differences were found on items of “Visual Engagement” *F*(2, 49) = 4.36, *p* = 0.018, “Behavior Engagement” *F*(2, 49) = 13.32, *p* < 0.001, “Social Engagement” *F*(2, 49) = 5.56, *p* = 0.007, and “Composite Sum” *F*(2, 49) = 4.13, *p* = 0.022 by ANOVA tests. Further post hoc examinations showed participants were significantly more engaged with C1 than CC in terms of “Visual” (*p* = 0.005), “Behavioral” (*p* < 0.001), and “Social” (*p* = 0.002) aspects of engagement and engagement in general (Composite Sum, *p* = 0.006). No significant difference was found between C1 and C2 on any rating items. Besides, significance was also exhibited on “Behavioral Engagement” between C2 and CC (*p* = 0.003), meaning that with the presence of either a toy or a robot, the behavioral aspect of engagement was reinforced.

No significant difference was found in OERS.

Regarding the PEAR–Apathy subscale, significant differences were found on the rating items of “Eye Contact” (Chi-square = 7.47, *p* = 0.024, *df* = 2), “Physical Engagement” (Chi-square = 22.80, *p* < 0.001, *df* = 2), “Purposeful Activity” (Chi-square = 22.06, *p* < 0.001, *df* = 2), and “Verbal Expression” (Chi-square = 8.28, *p* = 0.016, *df* = 2). Pairwise comparison results demonstrated a significant decrease in apathy-related behaviors when interacting with C1 compared to CC on “Eye Contact” (*p* = 0.023), “Physical Engagement” (*p* < 0.001), “Purposeful Activity” (*p* < 0.001), and “Verbal Expression” (*p* = 0.013). No significance was found on items “Facial Expressions” and “Verbal Tone”. Moreover, C2 had a significantly lower score upon the item “Purposeful Activity” than the control.

## 5. Discussion and Conclusions

### 5.1. Effects of Contextual Interactions on PWD

Four different rating scales demonstrated a number of consistent results: (1) the significant results discovered between the interaction with C1 and CC suggested that the contextual interactions, including HRI and multiple sources of feedback from both the robot (proximal interaction) and the augmented reality display (peripheral interaction), helped with capturing and maintaining PWD’s attention (such as visual gazing and physical manipulation) during engagement; however, did not contribute to provoking positive emotions or facilitating verbal communications; (2) the presence of either a proactive or static robot (robot On or Off) can help facilitate purposeful activities and motivate behavioral engagement; (3) participants held the least positive attitude towards the C2 compared to other experimental conditions, and this could be due to the childish feeling when playing with the turned-off robot. In conclusion, as the literature suggests, the notion of engagement is constructed by two essential components: affective state and focused attention [40]. The results of this study indicate the manipulation of the level of interactivity of a social robot and that the contextualized feedback positively influenced the attention aspects of engagement. As the literature suggested, customized content with reminiscent materials may contribute to enhanced affective engagement [28]. Future designs of HRI with PWD need to consider the properly designed interactivity of social robots, the role of contextual cues in HRI, as well as the content being conveyed through the context to enhance engagement from both affect and attention aspects of engagement. In addition, since most research on PWD focused on providing therapeutic stimulations through auditory and visual modalities, tactile interaction is also crucial for motivating interests in activity engagement, especially when other senses are compromised.

### 5.2. Reflections for Measurement Use

The adoption of four rating scales also generated several reflections on measurement use: (1) the combined use of OERS and OME can provide an effective overall evaluation of PWD’s engagement in terms of aspects of attention and affect/attitude; however, they do not address social or verbal engagement separately, which are useful parameters for social robot interaction evaluations; (2) EPWDS compensate above two scales by offering a separate evaluation of verbal and social engagement. Additionally, the rated scores of each item can be added up as a single sum for an easier comparison between conditions. However, one problem noted by authors is that the dimension “affective engagement” measures the extent to which the observer agrees that the participant displayed positive/negative affect, but it does not cover the frequency of emotional expressions and how long it lingers. This could be further improved or supported in use with OERS; (3) PEAR–Apathy is a valuable scale that assesses the extent to which the participants are intrinsically motivated to behave despite being positively or negatively engaged. It could be adopted in line with engagement evaluation for providing more a comprehensive understanding of user engagement.

### 5.3. Limitations and Future Works

The first limitation concerns the small sample size and data analysis. The sample size is relatively small due to challenges in participant recruitment and withdrawal during the experiment. Within each group of participants, a repeated measurement design was performed. However, we analyzed the data as between-subject instead of within-subject due to too many drop-out sessions, which consequently resulted in a loss of certain test power. The second limitation concerns the ethics of the social robot use in dementia care, as in certain situations, especially for people with severe stages of dementia, it may create a risk of deception due to the animal characteristics accompanied by environmental visual–auditory materials. Therefore, we insisted on a facilitator presence at all times for supervising the proper use. Future work will regard further statistical analysis of rating scale results over co-variables (e.g., dementia severity, limitation in language, emotion expression, and mobility), which could be tested to provide deeper insights. In addition, a full factorial experiment of the different levels of interactivity of the robot, and with/without the responsive context could be conducted to further investigate the influence of adding a context to HRI.

## Figures and Tables

**Table 1:** Three experimental conditions with descriptive details.

Variable	C1	C2	CC
Stimulus	The proactive robot: the robotic sheep responds to users’ stroke and touch by moving its head, neck, legs, and tail and making baby lamb sound.	The static robot: the robotic sheep was turned off; however, the tactile feature is still available and inviting to stroke and hug.	No physical stimulus.
Context	Reactive context: the virtual sheep in the screen display responds to users’ stroke and touch by being active and approaching user.	Dynamic context: the display plays looped video of the same content as in C1.	Dynamic context: same as in C2.

**Table 2:** Participant demographics including age, gender, type of dementia, marital status, stage of dementia, cognitive functions, wheelchair use, MMSE score, and length of stay in facility. No statistical difference was found between demographic characteristics of two groups of participants.

Characteristics	G1 *n* = 7	G2 *n* = 9	Total *N* = 16	*p* Value
**Age, mean (SD)**	86.6 (4.2)	84.1 (5.2)	85.2 (4.8)	0.325
**Female, n (%)**	5 (71.4)	7 (77.8)	12 (75.0)	0.789
**Type of dementia, n (%)**				0.717
Alzheimer’s Dementia	2 (28.6)	3 (33.3)	5 (31.3)	
Vascular Dementia	1 (14.3)	2 (22.2)	3 (18.8)	
Mixed Dementia	4 (57.1)	4 (44.4)	8 (50.0)	
**Marital status, n (%)**				0.598
Single/Divorced	1 (14.3)	1 (11.1)	2 (12.5)	
Married	4 (57.1)	4 (44.4)	8 (50.0)	
Widowed	2 (28.6)	4 (44.4)	6 (37.5)	
**Stages according to staff records, n (%)**				0.861
Mild	1 (14.3)	1 (11.1)	2 (12.5)	
Middle	2 (28.6)	3 (33.3)	5 (31.3)	
Middle to severe	3 (42.9)	2 (22.2)	5 (31.3)	
Severe	1 (14.3)	3 (33.3)	4 (25.0)	
**Cognitive functions reported by staff, n (%)**				0.877
Mild	1 (14.3)	2 (22.2)	3 (18.8)	
Confused at times	3 (42.9)	3 (33.3)	6 (37.5)	
Constantly confused	3 (42.9)	4 (44.4)	7 (43.8)	
**Wheelchair use, n (%)**	3 (42.9)	3 (33.3)	6 (37.5)	0.719
**MMSE score, mean (SD)**	14 (5.3)	11.3 (8.3)	12.88 (7.1)	0.475
Range	8–22	0–23	0–23	
**MMSE Stage, n (%)**				0.509
Stage 1 (>19)	2 (28.6)	2 (22.2)	4 (25.0)	
Stage 2 (10–19)	4 (57.1)	4 (44.4)	8 (50.0)	
Stage 3 (<10)	1 (14.3)	3 (33.3)	4 (25.0)	
**Length of stay, n (%)**				0.967
Six months or less	1 (14.3)	1 (11.1)	2 (12.5)	
More than 6 months	2 (28.6)	3 (33.3)	5 (31.3)	
More than 12 months	4 (57.1)	5 (55.6)	9 (56.3)	

Note: MMSE score above 19 were considered stage 1 of MMSE Stage, between 10–19 (including 10 and 19) were considered stage 2, and below 10 were considered stage 3.

## Appendix A

**Table A1:** Reported Cohen’s Kappa coefficient (*k*) to measure inter–rater reliability between two raters of all rating items of four observational rating scales.

Rating Scale Items		*k* Value
**OME**		
Attention	Most of the time	0.776
	Highest level	0.655
Attitude	Most of the time	0.685
	Highest level	0.675
**EPWDS**		
Affective Engagement		0.612
Visual Engagement		0.664
Verbal Engagement		0.719
Behavioral Engagement		0.678
Social Engagement		0.606
**OERS**		
Pleasure		0.782
General Alertness		0.642
Anger		1.000
Anxiety/Fear		0.755
Sadness		1.000
**PEAR–Apathy**		
Facial Expression		0.708
Eye Contact		0.649
Physical Engagement		0.750
Purposeful Activity		0.714
Verbal Tone		0.745
Verbal Expression		0.723

## Appendix B

**Table A2:** Kruskal–Wallis H tests with pairwise comparisons and ANOVA tests with post hoc examinations performed on all rating items of four observational rating scales to disclose the differences in engagement, affect, and apathy among experiment conditions: C1, C2, and CC.

Rating Scale Items		Conditions M (SD)			*p* Value			
		C1	C2	CC	Sig.	C1-CC	C2-CC	C1-C2
**OME**								
Duration (in seconds)		678.38 (244.94)	502.46 (266.38)	671.04 (372.91)	0.260	0.947	0.128	0.169
Attention	Most of the time	5.69 (0.95)	4.85 (0.99)	4.85 (1.19)	0.094	-	-	-
	Highest level	6.23 (0.83)	5.69 (1.03)	5.54 (0.95)	0.111	-	-	-
Attitude	Most of the time	5.46 (1.20)	4.31 (1.18)	4.62 (1.09)	**0.041**	0.125	1.000	**0.049**
	Highest level	5.92 (1.04)	5.00 (1.29)	5.19 (0.98)	0.094	-	-	-
**EPWDS**								
Affective Engagement		8.38 (1.50)	7.62 (1.50)	7.77 (2.42)	0.578	0.375	0.824	0.337
Visual Engagement		8.77 (1.69)	7.85 (1.82)	7.04 (1.73)	**0.018**	**0.005 ****	0.179	0.183
Verbal Engagement		8.23 (1.59)	7.85 (2.04)	7.54 (1.73)	0.517	0.257	0.612	0.583
Behavioral Engagement		8.85 (1.21)	8.00 (2.19)	6.58 (0.76)	**<0.001 *****	**<0.001** *******	**0.003 ****	0.118
Social Engagement		7.69 (1.55)	6.77 (1.69)	6.12 (1.14)	**0.007** ******	**0.002 ****	0.175	0.099
Composite Sum		41.92 (6.98)	38.08 (8.25)	35.04 (6.53)	**0.022**	**0.006 ****	0.213	0.173
**OERS**								
Pleasure		2.54 (0.97)	2.15 (0.69)	2.15 (0.93)	0.419	-	-	-
General Alertness		4.38 (0.77)	4.15 (0.80)	3.69 (1.02)	0.104	-	-	-
Negative Affect		3.38 (0.65)	3.54 (0.66)	3.42 (0.76)	0.669	-	-	-
**PEAR–Apathy**								
Facial Expression		2.15 (0.80)	2.69 (0.86)	2.69 (0.84)	0.120	-	-	-
Eye Contact		1.23 (0.44)	1.54 (0.78)	1.81 (0.63)	**0.024**	**0.023**	0.438	0.880
Physical Engagement		1.69 (0.75)	2.69 (1.18)	3.54 (0.71)	**<0.001 *****	**<0.001** *******	0.081	0.088
Purposeful Activity		1.62 (0.65)	2.46 (1.05)	3.42 (0.90)	**<0.001 *****	**<0.001** *******	0.043 *	0.191
Verbal Tone		2.08 (0.64)	2.46 (0.78)	2.42 (0.64)	0.345	-	-	-
Verbal Expression		1.46 (0.78)	2.00 (1.16)	2.35 (0.85)	**0.016**	**0.013**	0.574	0.542

Note: Bold values are * *p* < 0.05, ** *p* < 0.01, *** *p* < 0.001.
